# Compact simultaneous label-free autofluorescence multi-harmonic microscopy for user-friendly photodamage-monitored imaging

**DOI:** 10.1117/1.JBO.29.3.036501

**Published:** 2024-03-14

**Authors:** Geng Wang, Stephen A. Boppart, Haohua Tu

**Affiliations:** a University of Illinois at Urbana-Champaign, Beckman Institute for Advanced Science and Technology, Urbana, Illinois, United States; b University of Illinois at Urbana-Champaign, Department of Electrical and Computer Engineering, Urbana, Illinois, United States; c University of Illinois at Urbana-Champaign, Department of Bioengineering, Urbana, Illinois, United States; d Cancer Center at Illinois, Urbana, Illinois, United States; e University of Illinois at Urbana-Champaign, Carle Illinois College of Medicine, Urbana, Illinois, United States; f Center for Label-free Imaging and Multi-scale Biophotonics (CLIMB), Urbana, Illinois, United States

**Keywords:** multiphoton microscopy, nonlinear fiber optics, tunable ultrashort pulse, photodamage, label-free imaging

## Abstract

**Significance:**

Label-free nonlinear optical microscopy has become a powerful tool for biomedical research. However, the possible photodamage risk hinders further clinical applications.

**Aim:**

To reduce these adverse effects, we constructed a new platform of simultaneous label-free autofluorescence multi-harmonic (SLAM) microscopy, featuring four-channel multimodal imaging, inline photodamage monitoring, and pulse repetition-rate tuning.

**Approach:**

Using a large-core birefringent photonic crystal fiber for spectral broadening and a prism compressor for pulse pre-chirping, this system allows users to independently adjust pulse width, repetition rate, and energy, which is useful for optimizing imaging conditions towards no/minimal photodamage.

**Results:**

It demonstrates label-free multichannel imaging at one excitation pulse per image pixel and thus paves the way for improving the imaging speed by a faster optical scanner with a low risk of nonlinear photodamage. Moreover, the system grants users the flexibility to autonomously fine-tune repetition rate, pulse width, and average power, free from interference, ensuring the discovery of optimal imaging conditions with high SNR and minimal phototoxicity across various applications.

**Conclusions:**

The combination of a stable laser source, independently tunable ultrashort pulse, photodamage monitoring features, and a compact design makes this new system a robust, powerful, and user-friendly imaging platform.

## 1 Introduction

Intravital imaging technology with high speed, sufficient spatial resolution, and long-term photodamage-free capability is critical to the study of biological processes. It permits direct and longitudinal tracking of diverse intercellular behaviors in their native environment instead of inferring possible processes based on static images.^1^^–^^4^ As one type of intravital imaging, label-free nonlinear optical microscopy^5^^–^^10^ has become a powerful tool for biomedical research due to its advantages of low invasiveness, deep imaging penetration, and high resolution, etc., especially in neuroscience, oncology, and immunology.^11^^–^^15^ However, the relatively slow imaging speed and the accompanying photodamage risk present limitations to further preclinical and clinical studies. The improvement of imaging speed and the suppression of photodamage can be achieved by different methods, such as improving the excitation efficiency,^7^^,^^16^ using a polygonal mirror and resonant scanner,^17^ splitting one pulse to sub-pulses with equal energy,^18^ applying an adaptive light source to illuminate only the area of interest (ROI),^19^ improving the signal-to-noise ratio (SNR) of a single frame through a deep learning algorithm,^20^^,^^21^ or a heterodyne detection method.^22^^,^^23^ One fundamental factor that limits the imaging speed is the low excitation efficiency of a single pulse. For a typical 80 MHz imaging system,^24^ the power of each laser pulse has to be kept low to avoid heating-related photodamage. Therefore, to obtain an image with acceptable SNR, each pixel needs to contain tens to hundreds of pulses, which not only limits the imaging speed but also the low peak power makes it difficult to excite multiple nonlinear processes simultaneously.

In 2018, using a combination of a reduced repetition rate laser source (10 MHz) and shortened pulse width (<60  fs) at a 1110-nm central wavelength, You et al. obtained a relatively high peak power of optical pulses and demonstrated simultaneous label-free autofluorescence multi-harmonic (SLAM) microscopy,^7^ which can simultaneously acquire the signals of two- and three-photon excited autofluorescence (2PAF/3PAF) of FAD/NAD(P)H, respectively, and second/third harmonic generation (SHG/THG), by using the supercontinuum generation technology based on a photonic crystal fiber (PCF). However, several limitations remained: (1) The high peak power supercontinuum generation leads to a short life of the PCF (∼200  h), which needs to be replaced regularly and thus complicates the operation of the system; (2) The limited average power for a given excitation band (∼60  nm) does not allow users to adjust pulse repetition rate in a wide range to meet different imaging requirements; (3) Although the peak power of the excitation pulse is greatly improved, the photon-counting photomultiplier (PMT) detection requires a large number (>10, typically) of excitation pulses for each pixel (a long pixel dwell time) for sufficient signal (counts), which limits imaging speed; (4) The components used in the system are large, resulting in a rather bulky and complex system.

Here, we demonstrate a new and compact SLAM platform and system. The central wavelength of the excitation window is shifted to ∼1030  nm, which is more accessible commercially. By using a PCF and a prism compressor, we obtain excitation pulses from near-transform-limited 60 fs (FWHM) to uncompressed 300 fs with a broad bandwidth [∼80  nm (990-1070 nm), bottom-to-bottom], and sufficient pulse energy (or average power) under a wide and tunable repetition rate (800 kHz to 20 MHz). More importantly, these three pulse parameters (width, repetition rate, and energy) can be adjusted independently without interference. This allows users to find the optimal imaging conditions for different applications to maximize the signal-to-photodamage ratio. With only PCF-assisted spectral broadening free of supercontinuum generation, the laser source is stable indefinitely (>1 year) without the need to replace the PCF. Finally, the higher peak power permits single pulse per pixel label-free imaging via analog PMT detection, and can greatly increase the imaging speed paired with a faster optical scanner.

## 2 Materials and Method

For label-free nonlinear imaging, it is critical to achieve high excitation efficiency without photodamage. According to the nonlinear optical signal generation formula [Eq. (1)],^7^ when the average power P remains unchanged, the signal generation intensity S can be significantly increased by reducing the duty cycle, consisting of laser repetition rate f and pulse width τ, especially for high order nonlinear processes

$$S∼(f\tau ){\left[\frac{P}{f\tau }\right]}^{n}=\frac{P^{n}}{(f\tau {)}^{n-1}},$$ (1)

where n denotes the order of nonlinear process (2 for 2PAF/SHG, 3 for 3PAF/THG). For two/three-photon imaging, when the duty cycle is reduced by a factor of x, the signal will increase by a factor of x and $x^{2}$, respectively. Therefore, by reducing the duty cycle, equal or higher signal can be achieved using lower average power (lower risk of thermal damage^11^).

### 2.1 Excitation Window

For this new platform of SLAM microscopy, the central wavelength was shifted to 1030 nm. Compared with the previous 1110 nm center wavelength, the 1030 nm center wavelength has two main advantages: (1) The excitation center wavelength is the same as the source laser emission center wavelength for PCF spectral broadening, so there is no need to use high peak power to generate supercontinuum, and only the need to use low peak power to broaden the spectrum in the PCF to achieve a near-transform-limited pulse, which can avoid the problem of PCF damage and replacement; (2) Lower input peak power for PCF allows the same spectral broadening over a wide range of repetition rates (with a wide range of average power) to meet the needs of different applications.

### 2.2 Fiber Source

**Fig. 1:**
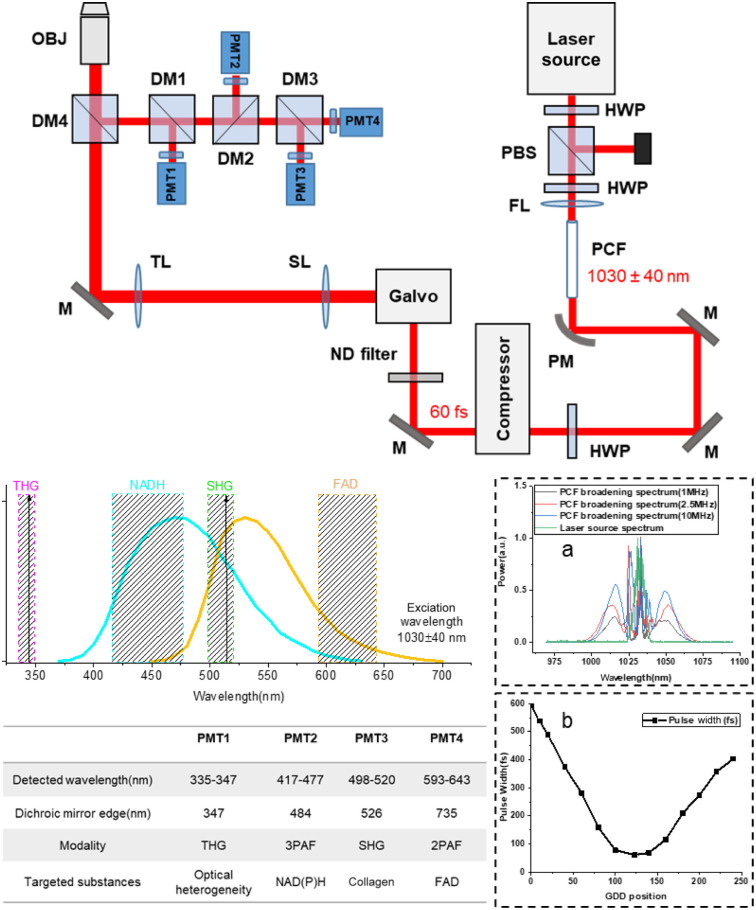
Schematic of the compact SLAM system with four spectral detection channels and the emission spectra of NAD(P)H and FAD. Inset a: spectra before (green) and after (black- 1 MHz, red- 2.5 MHz, blue-10 MHz) spectral broadening based on the birefringent PCF. High peak power pulses were sent into the PCF to broaden the spectrum. A prism compressor was used to compensate the dispersion to obtain near-transform limited pulses. Multimodal multiphoton signals were spectrally separated into four detection channels by long-pass dichroic mirrors and specific bandwidth filters and detected by PMTs as listed in the table. Inset b: Measured results of compressor GDD position and pulse width (for 2.5 MHz/180 mW input). HWP, half wave plate; PBS, polarizing beam splitter; FL, focal lens; PCF, photonic crystal fiber; PM, parabolic mirror; M, mirror; ND, neutral density; SL, scan lens; TL, tube lens; DM, dichroic mirror; OBJ, objective; PMT, photomultiplier tube.

The laser source for PCF spectral broadening is a compact ultrafast laser (Satsuma, Amplitude Laser) with 1030±5  nm central wavelength (inset a of Fig. 1), 10 W maximum average output power, <350  fs pulse width, and tunable repetition rate from single shot to 40 MHz. To obtain near-transform limited pulses to improve the multiphoton signal generation efficiency, laser pulses were first sent into a 25-μm-core PCF (LMA-25, Thorlabs) to achieve a coherent spectral broadening of around 80 nm (1030±40  nm, inset a of Fig. 1) with ∼80% coupling efficiency, and then the collimated beam was sent to a prism pulse compressor (BOA-1050, Swamp Optics) for dispersion compensation to obtain 60 fs near-transform limited pulses, which can be achieved by adjusting the group delay dispersion (GDD) via the compressor, as shown in the inset of Fig. 1(b). In practical applications, one should pre-chirp the pulse to compensate for the dispersion caused by the optical components after the compressor. Compared with the previous SLAM system,^7^ the new platform does not need to select an excitation window at 1110 nm in the supercontinuum, so this eliminates the need for the expensive pulse shaper. It should be emphasized that the spectral broadening in this paper is dominated by self-phase modulation while other nonlinear processes of supercontinuum generation (e.g., stimulated Raman scattering, four-wave mixing, soliton dynamics)^25^ can be safely neglected due to a low peak power input.

### 2.3 System Setup

We designed a new SLAM platform and system (Fig. 1). The 1030±40  nm pulses from the prism compressor were raster scanned by a 4 mm aperture Galvo-Galvo Scanner (LSKGG4, Thorlabs) and focused by an inverted high UV-transmission objective (UAPON 40XW340, N.A. = 1.15, Olympus) with an ample adjustable average power on the sample after the loss along the excitation beam path. One telecentric scan lens (SL50-2P2, f=50  mm, Thorlabs) and one tube lens (TTL200MP, f=200, Thorlabs) expanded the beam size to fill the back focal plane pupil of the objective (Ø10.35  mm). Since high power is not required to generate supercontinuum, this system with its PCF can operate over a wide range of repetition rates (800  KHz∼20  MHz) with similar spectra broadening, as shown in the inset of Fig. 1(a) (1/2.5/10 MHz). For example, when the repetition rate is 2.5 MHz, the excitation power before the PCF is only 180 mW, but the maximum average power on the sample can exceed 50 mW, which gives users a high power dynamic range for different applications. Based on the objective lens we use, the typical maximum field of view (FOV) is around 350  μm×350  μm, the actual FOV depends on the scanning angle of the galvanometer-driven mirror. Due to the use of many integrated components, the dimension of the current system is about 1.5  m×1.7  m on a single-layer optical breadboard plate, which is about one-third of the previous SLAM system.^7^ Therefore, it is feasible to build it on a cart with multiple layers of plates.

### 2.4 Channels for Detection

Regarding the detection part, this system is designed to detect NAD(P)H from the 3PAF channel and FAD from the 2PAF channel, combined with noncentrosymmetric structures from the SHG channel and interfacial features from the THG channel, all simultaneously. The reflected multimodal multiphoton signals were spectrally-separated into four detection channels by long-pass dichroic mirrors and appropriate band-pass filters, as shown in Fig. 1. To minimize the crosstalk between individual channels and maximize the detection efficiency of each channel, the four channel filters were chosen with specific bandwidth characteristics and listed in Fig. 1. For this system, the high pulse peak power can enable more than one photon excitation per pulse when operating at low repetition rates. Therefore, the spectrally-separated signals were detected by analog PMTs instead of photon-counting PMTs (PMT2101, Thorlabs for 3PAF/ 2PAF/ THG channels; PMT1001, Thorlabs for SHG channel).

### 2.5 Imaging Acquisition and System Performance

To make the operation of this system more user-friendly, commercial control software (Scanimage, Vidrio Technologies, LLC) was used, which has powerful features and can allow users to set different scanning schemes by customizing key parameters, such as the number of pulses per pixel, FOV, repetition rate, etc. The details of the configuration control interface can be found in Ref. 26. For this compact SLAM platform, since the sampling clock is synchronized with the fundamental clock of the laser source, the number of pulses per pixel can be set via the pixel bin factor (single/multi-pulse), which is limited by the line period (cannot exceed the maximum scanning rate of the Galvo scanner) and also related to image pixel size. The frame rate is related to the line period and image pixel size, and the pixel dwell time is set by a combination of the repetition rate and pixel bin factor. Furthermore, since a pulse picker is used in this laser to adjust the repetition rate, the tunable repetition rate should be divisible by the fundamental rate of laser source (40 MHz). When the repetition rate is 0.83 MHz and the image size is 1024×1024  pixels, one pulse per pixel can be set at a frame rate of 0.7 Hz. Similarly, a minimum of 2/3/6/12/24 pulses per pixel can be set at 1.67/2.5/5/10/20 MHz repetition rate, and the number of pulses per pixel can be increased by reducing the scanning speed of the Galvo scanner. In summary, the frame rate, line rate, pixel dwell time and average power on sample are dependent parameters. Once the repetition rate, pulse energy, bin factor, and image pixel size are fixed, these dependent parameters are also determined. The main independent parameters of this system are shown in Table 1.

**Table 1:** Independent parameters of the compact SLAM.

Typical repetition rates (MHz)	0.83, 1.67, 2.5, 5, 10, 20
Typical pulse width (fs)	60∼300
Pulse energy (nJ) or average power (mW) on sample	<5 or <20 (limited by photodamage)
Central wavelength (nm)	∼1030 (fixed)
frame size (pixel)	1024 × 1024 (fixed)
Number of pulses per pixel (bin factor)	1–24 pulses
Data acquisition along fast scanning direction	Bidirectional
Zoom factor (or pixel size)	Diffraction-limited sampling (or fixed FOV of 350 μm×350 μm)
Objective (magnification, NA)	40×, 1.15 (fixed)
Imaging depth (μm)	<200 (limited by working distance of the objective)

Altogether, by using a 25  μm core PCF and prism pulse compressor, we obtained excitation pulses from near-transform-limited 60 fs to uncompressed 300 fs with broad bandwidth (∼80  nm), and sufficient average power (or pulse energy) under a wide and tunable repetition rate (800 kHz to 20 MHz). More importantly, these three parameters can be adjusted independently without interference. This allows users to find the optimal imaging conditions for different applications. To distinctly delineate the primary distinctions and enhancements between this compact SLAM system and our group’s previous SLAM,^7^ we tabulated a comparative summary in Table S1 in the Supplementary Material, encompassing key aspects such as the laser source, detection system, performance, and applications.

## 3 Experimental Results and Discussion

### 3.1 *Ex Vivo* Lable-Free Mouse Tissue Imaging With One Pulse per Pixel Imaging Capability

**Fig. 2:**
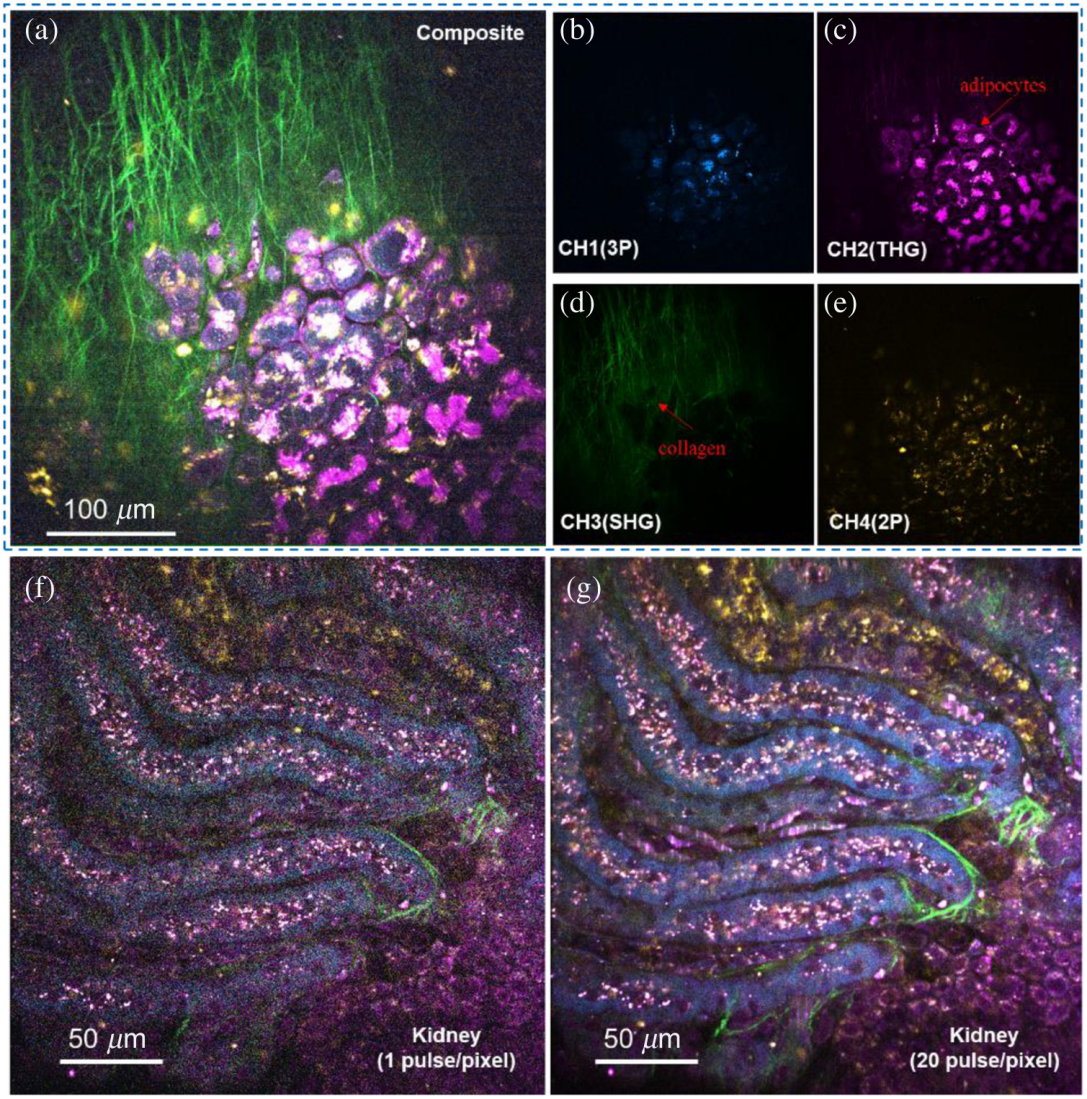
*Ex vivo* label-free imaging of mouse tissue by the compact SLAM system. Color in this figure: cyan (3PAF), magenta (THG), green (SHG), and yellow (2PAF). (a) Imaging of the inner surface of the mouse skin with four-channel composite information. (b) 3PAF signals with NAD(P)H information, (c) adipocytes revealed in THG signals. (d) Collagen fibers revealed in SHG signals, (e) 2PAF signals with FAD information, (f) single-frame imaging of a mouse kidney (composite) at one excitation pulse per pixel. (g) Image averaged over 20 frames under the same imaging condition as in (f).

Unstained and unlabeled mouse tissue was used to demonstrate the imaging capabilities of this system, as shown in Fig. 2. Among them, Figs. 2(a)–2(e) were acquired at a repetition rate of 2.5 MHz with 10 mW average power on the sample. The FOV is around 350  μm×350  μm with 1024×1024  pixels per frame and three pulses per pixel in one single frame. The images shown were obtained by averaging 10 frames with 0.7 Hz single frame rate (1024×1024  pixels per frame). It is worth mentioning that the current frame rate is limited by the relatively slow scan rate of the Galvo-driven mirror (500 Hz maximum), which can be addressed by using a high-speed rotating polygonal mirror or a resonant scanner. The images within the dashed box were the fat-rich areas on the inner surface of the mouse skin [Figs. 2(a)–2(e)]. The rich information of four different modalities is clearly reflected through the four different spectral channels. Among them, channel 1 is the 3PAF signal representing NAD(P)H information [Fig. 2(b)], channel 2 is the THG signal [Fig. 2(c)], channel 3 is the SHG signal [Fig. 2(d)], and channel 4 is the 2PAF signal representing FAD information [Fig. 2(e)]. Clear adipocytes and cell membranes are evident in the THG channel, and abundant fibers are observed in SHG channel. The composite image with four different modalities is shown in Fig. 2(a). Because the shift in central wavelength impacts excitation efficiency of sample, this experiment confirmed that FAD and NAD(P)H can still be simultaneously excited at this new wavelength using the new laser source and detected using new probe configuration. Importantly, the compact system accomplishes this without necessitating exceptionally high power for supercontinuum excitation.

The label-free multi-channel imaging capability with a single excitation pulse per image pixel was also demonstrated through the sample of *ex vivo* mouse kidney, as shown in Figs. 2(f) and 2(g), which were collected at a repetition rate of 0.83 MHz with 3.6 mW power on the sample and comprise the information of four channels (2PAF, 3PAF, SHG, and THG). The FOV is around 250  μm×250  μm with 1024×1024  pixels per frame. Figure 2(f) is a single-frame imaging that contains one pulse per pixel, and Fig. 2(g) (20 pulses per pixel) is the result of averaging 20 frames under the imaging conditions of Fig. 2(f). Comparing the two images, it can be seen that the single-frame imaging with a single pulse per pixel can reveal the tissue structure with a relatively low SNR [∼22% of Fig. 2(g)]. If paired with a high-speed scanner, the system could achieve high-frame-rate, label-free, long-term imaging with a low risk of nonlinear photodamage.^27^ This combination not only enhances imaging speed but also reduces the risk of photodamage, making it a valuable approach in the field.

### 3.2 Photo-Damage Monitored Imaging

Photodamage is a common problem in the field of optical bioimaging and is considered throughout different imaging techniques.^28^^–^^31^ This compact SLAM system with tunable ultrashort pulses can generate enough power on the sample to allow users to find the optimal imaging conditions for different applications based on monitored-photodamage limits and SNR.

**Fig. 3:**
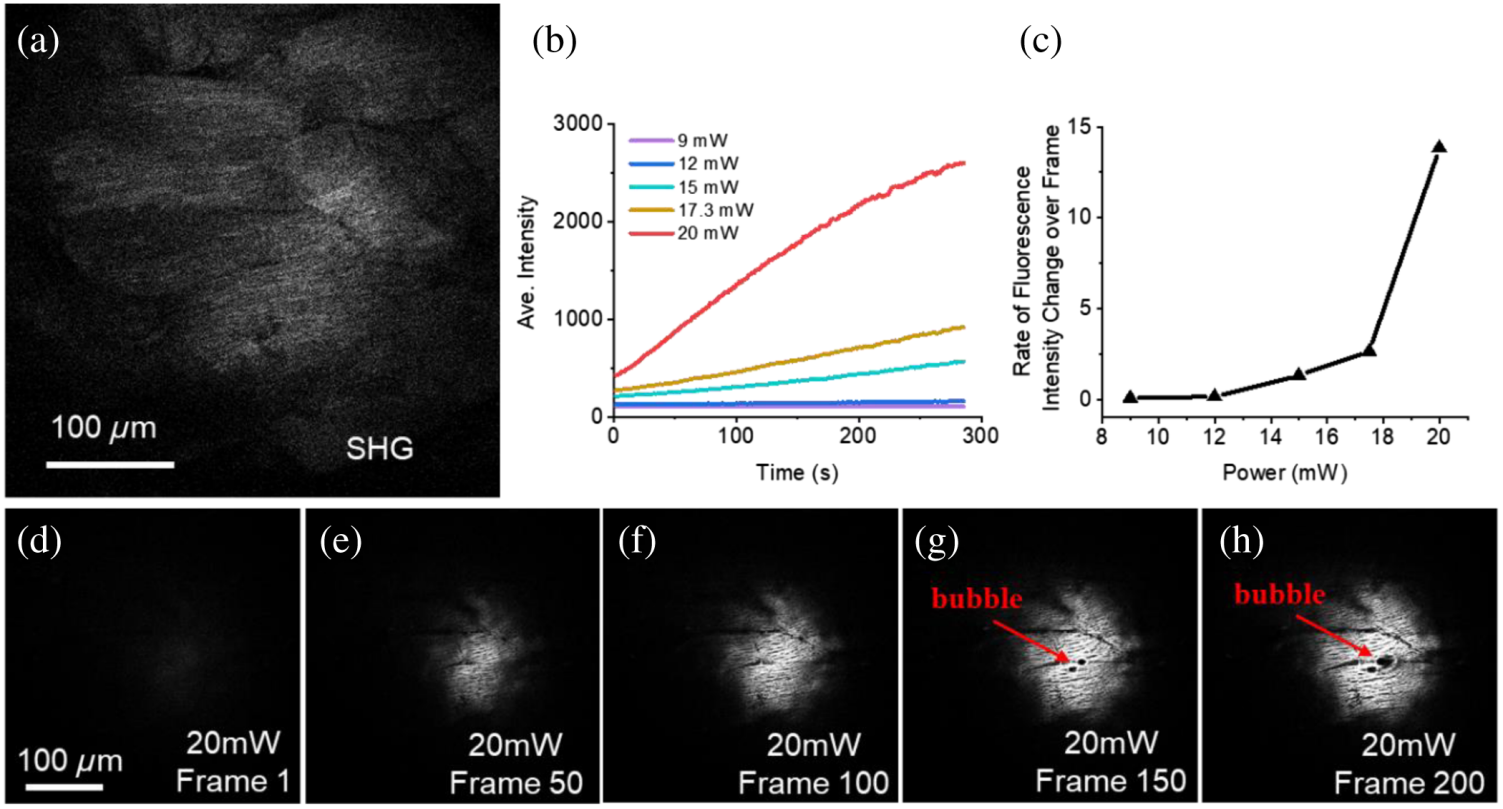
Photo-damage monitored imaging in tissue by the compact SLAM. (a) SHG channel was used to observe the muscle structure of the chicken breast to find the ROI for photo-damage experiment. (b) Changes in the average intensity of three-photon channel images of chicken breast over time under irradiation power of 9, 12, 15, 17.3, and 20 mW. (c) Three-photon signal growth rate as a function of incident power. (d)–(f) The 1st, 50th, 100th, 150th, and 200th three-photon channel images of chicken breast under 20 mW irradiation, which correspond to the red line in (b).

Because of its high spatial homogeneity, *ex vivo* chicken breast was used in this experimental study of photodamage. By adjusting the power on the sample, we observed the photodamage of the sample by time-lapse imaging. For the homogeneous chicken breast tissue, almost no autofluorescence signal was observed in the three-photon channel without photodamage. However, we can observe the structure of muscle tissue through the SHG channel for focusing and determining the ROI, as shown in Fig. 3(a).

We found that the 3PAF signal does not change in fluorescence intensity over time (200 frames in total, 285 s) when the power was low (9 mW on sample/2.5 MHz/60 fs), as shown in Fig. 3(b) (purple line). With the increase of the power on sample [9  mW∼20  mW, Fig. 3(b)], some newly generated fluorescent signals were observed in the 3PAF channel first, increased over time until bubbles were generated, and spread from the center to the edge gradually after more exposure, as shown in Figs. 3(d)–3(h) (20 mW on sample/2.5 MHz/60 fs). The new generated fluorescent signals were caused by the emergence of new substances due to photodamage^29^ and were mainly induced by photoionization process, while the subsequent bubbles were generated by the combination of photothermal and photoionization processes indicative of a more severe damage. Moreover, the rate of fluorescence intensity change over the frame number in the 3PAF channel behaves nonlinearly with increasing incident power [Fig. 3(c)]. Because different samples have different damage thresholds, by changing the power, repetition rate, and pulse duration, the users can find optimal imaging conditions to maximize the signal-to-photodamage ratio for their applications.

### 3.3 Denoising Using Time-Gated Window

**Fig. 4:**
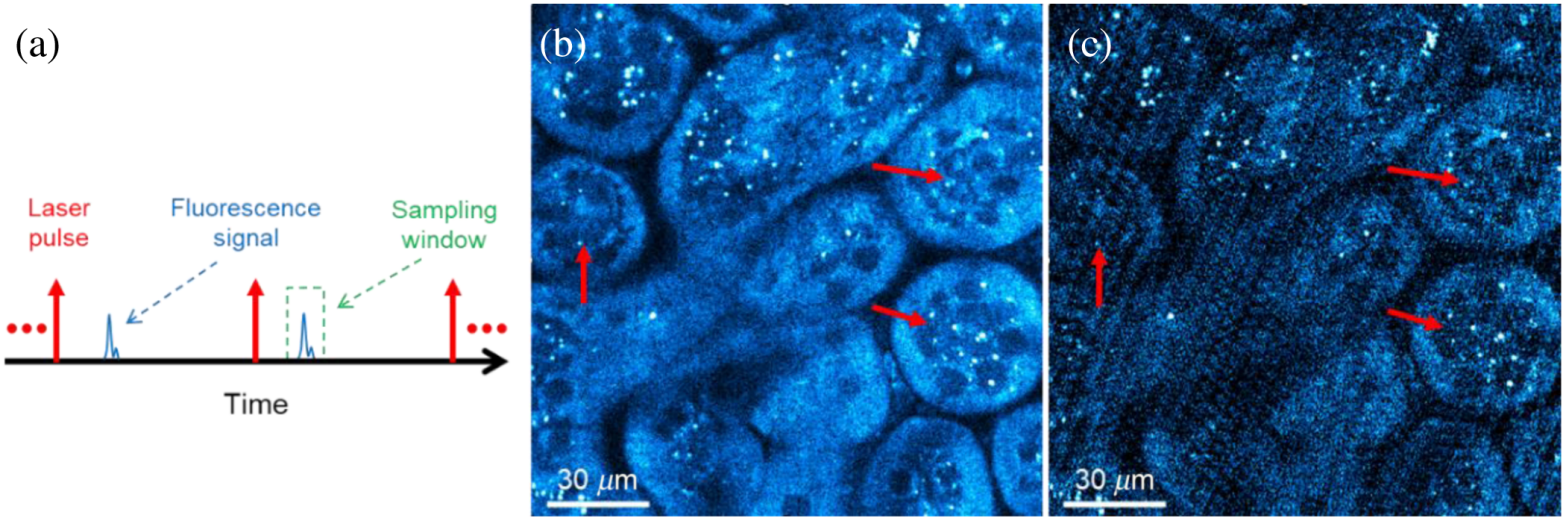
Noise reduction using time-gated window. (a) Schematic of time-gated window. (b) Three-photon mouse kidney image with a 10 ns sampling window for 5 MHz system. (c) Three-photon mouse kidney image with a 100 ns sampling window for 5 MHz system.

For laser scanning fluorescence imaging, the signal occupies only a small part between laser pulses, and the rest is useless noise, especially for the low repetition rate laser imaging system, as shown in Fig. 4(a). Since each pixel represents the average fluorescence signal integrated over the dwell time of that pixel, integrating these noisy regions within the signal can greatly reduce the SNR of the image, especially for the weak signal imaging. To reduce this adverse effect, the system applied a time-gated sampling window for each laser pulse through the data acquisition hardware to extract the useful fluorescence signal. An *ex vivo* mouse kidney sample was imaged to illustrate the effect of applying time-gated windows with different durations, as shown in Fig. 4(b) (10 ns) and Fig. 4(c) (100 ns), and the time interval between two laser pulses was 200 ns (5 MHz repetition rate) for this test. Compared with the image with the shorter duration sampling window [Fig. 4(b)], the scheme for using a longer duration sampling window [Fig. 4(c)] or integrating over the whole region greatly reduced the SNR (∼10  dB) and detection limit of the image, resulting in weak signal regions being buried in the noise, as shown in the areas indicated by red arrows in Figs. 4(b) and 4(c). The linear noise in Fig. 4(c) is caused by the electronic noise of the PMT, which can also be suppressed by using a time-gated window. As for the duration setting of the sampling window, it should be longer than the fluorescence lifetime of the sample. Furthermore, since the quality of noise suppression will affect the quantification of multiphoton imaging results, we also verified the excitation performance and linear correlation of this system through power and concentration-dependent experiments, as shown in the Supplementary Material.

### 3.4 Discussion

Benefiting from the innovative laser light source proposed in this study, we demonstrated single-pulse single-pixel label-free imaging at a repetition rate of 0.83 MHz, but imaging at a frame rate of 0.7 Hz is mainly limited by the low scanning rate Galvo-driven mirror (generally <500  Hz). Since we can produce a high repetition rate output without changing the peak power, if pairing a high scan-rate resonant mirror (several kHz) with a high repetition rate laser output (5 to 10 MHz), high frame rate label-free dynamics imaging can be achieved. Given that different samples possess varying damage thresholds, users can leverage their system’s capabilities by adjusting the power, repetition rate, and pulse duration. This empowers users to identify optimal imaging conditions that maximize the signal-to-photodamage ratio for their specific applications. In practice, for general tissue imaging, the damage threshold is within a certain range, which can be known through preliminary tests, and there is no need to conduct damage experiments on the tissue to be tested every time. If long-term experiments on a certain type of tissue or cell are required, the optimal imaging conditions can be found in the first test of the sample, and this condition can be used for subsequent tests. In terms of imaging quantification, although the noise problems of low repetition rate imaging systems can be reduced by using time-gated windows, the spectral cross talk between different channels cannot be ignored. However, this challenge can be addressed by initially establishing a cross-talk matrix between different channels through imaging of the target sample, and subsequently employing the mathematical method of inverse matrix to eliminate the cross talk. Also, the minimum time-gated window of the system is determined by the sampling rate of the hardware. When using a digitizer with several GHz sampling rates, fluorescence lifetime imaging can be achieved simultaneously. Prior to commencing daily imaging procedures, it is advisable to conduct quality checks on the laser beam using a beam profile following the pulse compressor and utilizing a spectrometer to verify spectral broadening. This precautionary measure serves to mitigate potential disruptions caused by the change of laser source, ensuring the consistency and reliability of long-term quantitative biological studies. In addition, the system’s high peak power hints at the potential for higher-order nonlinear imaging, an avenue deserving of future exploration.

## 4 Conclusion

We built a compact SLAM system based on off-the-shelf optical and mechanical components, which enabled simultaneous excitation and acquisition of 2PAF/3PAF from FAD/NAD(P)H and SHG/THG signals via a single-excitation source. The high excitation efficiency enabled by the higher peak power improved the image SNR from a single pulse. By using a robust 25  μm large-core PCF and prism compressor, we obtained excitation pulses from near-transform-limited 60 fs to uncompressed 300 fs with broad bandwidth (∼80  nm), and sufficient average power under a wide and tunable repetition rate (800 kHz to 20 MHz), and realized one pixel per pulse label-free imaging. Moreover, pulse repetition rate, pulse energy, and pulse duration on the sample can be adjusted independently without interference to satisfy different applications. Daily operation of this imaging system has been reliable, as imaging can be routinely performed 10 min after the laser is turned on. The occasional drift in fiber coupling alignment can be easily compensated by attaining the same spectral broadening with the same input laser power. Finally, the compact size allows the whole microscope to be integrated into a movable cart for clinical study. These advantages make this new and compact SLAM system a powerful and user-friendly imaging platform.

## Supplementary Material



## Data Availability

The code and data presented in this manuscript can be made available from the corresponding author through a collaborative research project.
