# Supplementary material for: Compact simultaneous label-free autofluorescence multi-harmonic microscopy for user-friendly photodamage-monitored imaging
Source: J Biomed Opt. 2024 Mar 14;29(3):036501. doi: 10.1117/1.JBO.29.3.036501 (PMC10939229; doi:10.1117/1.JBO.29.3.036501)
Supplement: Supplementary file 1 [file JBO_029_036501_SD001.pdf]

Supplemental Material

Table S1 Distinctions and enhancements between compact SLAM and SLAM system.

	**SLAM** [7]	**Compact SLAM**
Excitation wavelength	1110±30 nm	1030±40 nm
Repetition rate/ Power before PCF	10 MHz/ 3.5 W	0.83- 20 MHz/ 0.05-1.2W
Supercontinuum generation	Yes	No
PCF diameter (type, vendor) /lifetime	15 µm (LMA-PM-15, Thorlabs) / 200 hrs	25 µm (LMA-25, Thorlabs) / >1 year
Frame rate	0.5 Hz	0.7 Hz (higher with high speed scanner)
PMT	Photon-counting PMT	Analog PMT
Compressor (vendor)	MIIPSBox640 (Biophotonic Solutions)	BOA (Swamp Optics)
Control software	Labview-based(customer)	Matlab-based(commercial)
Size	3.5 m ×1.5 m	1.7 m ×1.5 m
Tunable parameters	—	repetition rate, power on sample and pulse duration can be adjusted independently without overlapping interference.

Supplemental Note

For the low repetition rate laser imaging system, integrating all the region between two pulses as a signal affects the real signal as described in 3.3, especially in the case of low signal generation, because the signal may be swamped by noise. To verify the multiphoton excitation performance of this compact SLAM system, power-dependent experiments were performed using time-gated window based on 0.83 MHz repetition rate. If we conduct logarithm operation on both sides of *Eq. 1*, we obtain:

$$\log_{10} S = n \cdot \log_{10} P + (1 - n) \cdot \log_{10}(f\tau) \quad \text{(S1)}$$

where the second term on the right side of the *Eq. S1* is a constant. If $(1-n)\cdot\log_{10}(f\tau)$ is replaced by c, $\log_{10} S$ is replaced by y, and $n\cdot\log_{10} P$ is replaced by x, *Eq. S1* can be simplified to *Eq. S2*:

$$y = n\cdot x + c \tag{S2}$$

Therefore, through power dependent experiments and linear fitting, the nonlinear order- n of each channel can be measured. For this system, to perform power dependent experiments, an FAD solution was used for the 2PAF channel, an NADH solution was used for 3PAF channel, and the surface signal of a coverslip was used for the THG channel, as shown in Fig. S1(a)- S1(c). The experimental results showed that the linear fitting slope of the two-photon channel is ~2(2.017), and the linear fitting slope of the three-photon and THG channels is ~3(2.984 and 3.027), which is consistent with the theory. Meanwhile, solution concentration-dependent experiments were also performed to validate the linear correlation between the concentration and intensity based on an analog PMT, as shown in Fig. S1(d). The results indicate that solution concentration and signal intensity exhibited a proportional linear relationship without signal saturation. Furthermore, based on the proportional linear correlation between the signal intensity and concentration of autofluorophore, the concentration of the multiphoton signal can be quantified based on the calibrated power/concentration dependent experiments, even for the analog PMT.

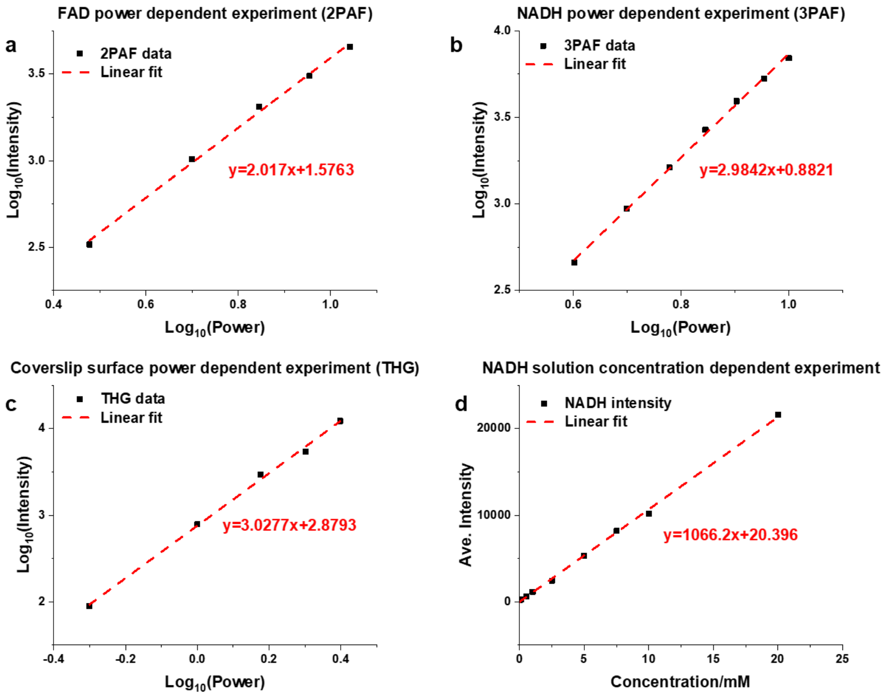

Fig. S1 Power and concentration dependent experiments on the compact SLAM system. (a) 2-photon channel power dependent experiment (3,5,7,9,11 mW on sample) based on 10 mM FAD solution. (b) 3-photon channel power dependent experiment (4/5/6/7/8/9/10 mW on sample) based on 10 mM NADH solution. (c) THG channel power dependent experiment (0.5/1/1.5/2/2.5 mW on sample) based on coverslip surface. (d) 3-photon channel concentration dependent experiment (0/0.1/0.5/1/2.5/5/7.5/10/20 mM) of NADH solution with 4 mW on sample.
